# A single-phase direct buck-boost AC–AC converter with minimum number of components

**DOI:** 10.1038/s41598-023-35770-9

**Published:** 2023-06-02

**Authors:** Fawzy Adel, Azza E. Lashine, Awad E. El-Sabbe, Dina S. M. Osheba

**Affiliations:** Electrical Engineering Department, Faculty of Engineering, Menoufia University, Shebin El-Kom, 32511 Egypt

**Keywords:** Electrical and electronic engineering, Energy infrastructure

## Abstract

In this paper, a single-phase direct pulse width modulation (PWM) buck-boost AC–AC converter is proposed. The proposed converter utilizes a minimum number of semiconductor switches and passive components that decreases the converter power losses and offers high efficiency. It can be operated with simple PWM control and doesn’t require soft-commutation strategies. It does not suffer from input source shoot-through and commutation problems. Moreover, it supplies both continuous input and output currents. The common sharing ground of the input and output gives the proposed converter the feature that it can be utilized for voltage sag and swell compensation. A comparison of the proposed converter performance with similar existing converters is presented. Also, detailed circuit analysis, component design guidelines, and simulation results using the MATLAB/Simulink environment are demonstrated. A laboratory prototype has been built and tested to validate the converter performance and confirm the results obtained by computer simulation.

## Introduction

The AC–AC converters have a sensitive role in industry as they are commonly utilized in some industrial applications, such as induction machine adjustable speed drives, dynamic voltage restorers (DVRs), compensation of voltage sag and swell, lightning control, and electric heaters. Due to their vital role, there is a continuous study and a growing interest in the development of AC–AC converters. Traditionally, the AC–AC conversion was achieved by the AC thyristor controllers that employ the phase angle control to get the desired output voltage. These circuits suffer from many drawbacks, such as low input power factor, large total harmonic distortion, low efficiency, and the need for large passive filters^1–5^.

The PWM converters introduced in^6–11^ overcome the drawbacks of the AC thyristor controllers as they provide a method to solve the commutation problems and offer higher efficiency than the AC thyristor controllers. In^6^, a PWM converter is introduced that offers inverting and non-inverting output voltage and the commutation problems are solved by using the switching cell structure and coupled inductors. The converter introduced in^7^ is a modification for that introduced in^6^. It is implemented by using the Z-source impedance that offers a safe commutation and provides a wider range of the output voltage. Moreover, the converters introduced in^8–11^ are a modification of those introduced in^6,7^, and also, they are immune from commutation problems. In^8^, they eliminate the filter inductor from the switching cell AC–AC converters by using magnetic integration. In^9^, a number of separate units of switching cell structure multilevel AC–AC converter are connected in series to attain high voltage levels. Also, the use of switching cell structure multilevel AC–AC converter as a dynamic voltage restorer is introduced in^11^.

In recent years, the pulse width modulation (PWM) AC–AC converters have already attracted a lot of attention, mainly due to their high efficiency, simple structure, better power factor, lower harmonics, ease of control and smaller input/output filter requirements compared to the AC thyristor controllers.

The PWM AC–AC converters are divided into three categories: direct AC–AC converters, indirect AC–AC converters, and matrix converters. Both the indirect and matrix converters can adjust both the output voltage and frequency. The indirect AC–AC converters have a two-stage power conversion of AC–DC and DC–AC. In addition, they need a large DC-link capacitor and filter inductors, which increase their size, cost, and power losses^12–15^. The matrix converters require a higher number of semiconductor switches, leading to low efficiency and high size and cost. Moreover, it suffers from severe commutation problems due to the dead time and overlap time between the semiconductor switches^16–19^. The direct AC–AC converter is a single-stage power conversion that can adjust the output voltage without any need for the bulky short-life DC-link capacitor, thus it is preferred for applications that require only a regulation in the output voltage due to its single stage conversion, smaller size, high efficiency, and low cost^1,5,20^.

The traditional direct AC–AC converters have been developed from the traditional DC–DC converters by replacing the unidirectional switches with bidirectional switches. They also include buck, boost, buck-boost and Cuk converters^21–23^. All of these topologies suffer from commutation problems due to the overlap-time and dead-time between the complementary switches. The overlap and dead time cause current and voltage spikes that damage the semiconductor switches.

Another category of AC–AC converters is the impedance-source (Z-source) AC–AC converters. The Z-source concept was extended to AC–AC conversion for the first time in^2^. The Z-source AC–AC converter introduced in^2^, can buck and boost the input voltage by using the impedance source network, but it suffers from commutation problems. A family of conventional Z-source AC–AC converters is introduced in^3^ and they can buck and boost the input voltage and require a complex switching strategy as a method for solving the commutation problems. In^4,5^ a quasi-Z-source and a modified quasi-Z-source AC–AC converter were developed to overcome the drawbacks of the conventional Z-source AC–AC converter in^2,3^.

In^24^, single-phase direct AC–AC converters are implemented by replacing the bidirectional switches of the traditional PWM AC–AC converters with the switching cell structure and the coupled inductor. Although this method solves the commutation problem and eliminates the reverse recovery problems, these converters suffer from circulating current components that increase conduction loss, switching loss, and current stresses for the switching devices in the converter, which lead to efficiency degradation. In^25^, a group of single-phase direct PWM AC–AC converters are designed to overcome the disadvantages of the SC AC–AC converters in^24^. They eliminate the circulating currents and decrease the magnetic volume of the coupled inductors by replacing the coupled inductors with small inductors. However, they also use a complementary PWM strategy, so benefits such as lower input and output harmonics, lower THDs, and smaller size of the required filters were lost. The use of the switching cell structure in^24,25^ increases the number of switches and passive components that increase the converter size and power losses.

A single-phase buck-boost AC–AC converter is introduced in^26^. It requires two inductors, four switches and four external diodes. This converter is operated by a complex commutation strategy and requires two capacitors to provide current paths during dead time and to solve commutation problems.

In^27^ a Z-source AC–AC converter is obtained by replacing the inductors of the converter of^26^ with the coupled -inductor-based Z-source impedance networks. This converter is implemented by four semiconductor switches and four diodes. It also requires two capacitors, each of them connected across two switches to reduce the commutation spikes. All the above converters require a large number of semiconductor switches and passive components that increase the converter’s size and cost and decrease its efficiency.

The two converters in^28,29^ have high efficiency, quasi-continuous input and output currents, and a lower size of reactive components. Despite their advantages, the two converters employ a high number of semiconductor switches and a high number of reactive components, as they are implemented by two main inductors, two main capacitors and six semiconductor switches. In addition to a high number of switches operated with high frequency in each mode of operation that increasing the circuit losses.

A single-phase direct-buck AC–AC converter for grid voltage compensation is introduced in^30^, which needs a large number of components. Six semiconductor switches, six diodes, one inductor, and one capacitor are used in this topology. The direct AC–AC converter also can be utilized as a line-frequency-isolation flexible AC-link converter for voltage compensation and power flow control, as introduced in^31^. There are many studies are carried out to achieve better controllability and flexibility in AC–AC converters. In^32^, an arbitrary two-phase input voltage based AC volage synthesis method and the corresponding modulation strategy for direct AC–AC power conversion is introduced. By using this method, the frequency, amplitude, and phase modulation for direct ac–ac power conversion are realized.

In this paper, a single-phase direct AC–AC converter that can operate as a voltage buck-boost converter is proposed. The proposed converter requires a minimum number of switches (four switches), and a minimum number of passive components (one inductor and one capacitor). In each mode of operation, there is only one switch that operates with the body diode of another switch, which decreases the circuit losses. The proposed converter doesn’t suffer from any commutation problems, as the supply current is continuous and there is a path for the supply and inductor currents in all times. Also, there isn’t any risk of the input source shoot through even if all switches are turned on at the same time. Therefore, it doesn’t require complex safe-commutation strategies and operates with a simple switching control. The common sharing ground provides the feature to measure the voltage difference between the input and output voltages and then detect the voltage variation due to voltage sags or swells. This is a critical feature of proposed AC–AC converter that enables it to provide voltage regulation and compensation for voltage sag and swell.

## The proposed converter

### Circuit topology

**Figure 1:**
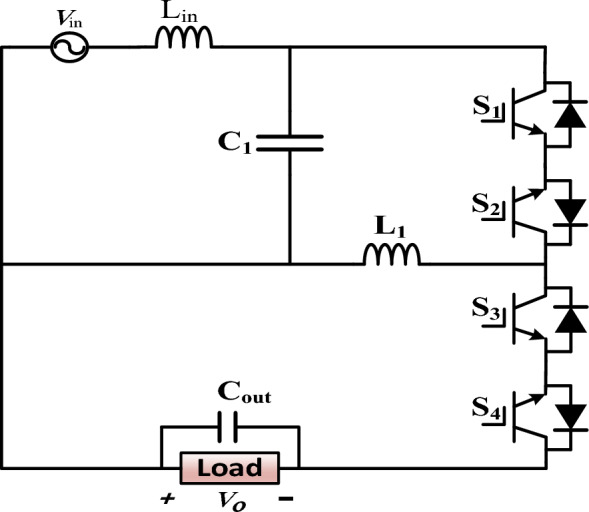
Proposed single-phase direct buck-boost AC–AC converter.

The circuit configuration of the proposed direct buck-boost AC–AC converter is shown in Fig. 1. It is implemented with four switches (S_1_–S_4_), one inductor (L_1_), and one capacitor (C_1_). The required input inductor and output capacitor filters are represented by L_in_ and C_out,_ respectively.

### Switching strategy

**Figure 2:**
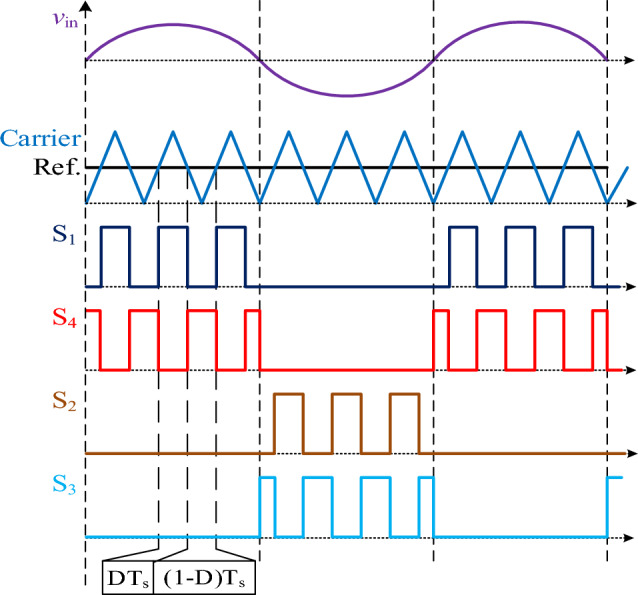
Gating signals of the proposed converter.

The gating signals are generated by a conventional carrier-based pulse-width modulation (PWM) method, as shown in Fig. 2. Where D is the duty cycle and T_s_ is the switching time period.

The PWM signal goes to the gates of the switches S_1_ and S_2_, while its complementary goes to the gates of S_3_ and S_4_, as shown in Fig. 2. There are two modes of operation during each half cycle of the input voltage. There is only one switch ON, and the body diode of another switch is forward biased during each mode of operation. Therefore, it has a continuous current waveform that indicates high current quality.

### Modes of operation

**Figure 3:**
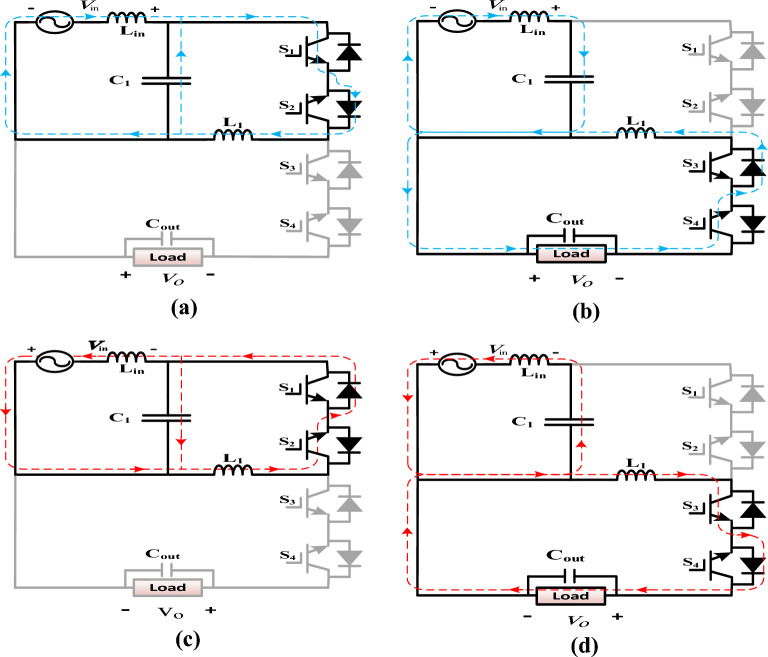
Modes of operation of the proposed AC–AC converter (**a**) Mode 1, (**b**) Mode 2, (**c**) Mode 3, (**d**) Mode 4.
  - Mode 2 [DTs-Ts]:
    During this mode of operation, the switch S_4_ is turned-on, and the body diode of S_3_ is forward biased for an interval (1-D)T_s_ as shown in Fig. 3b. The energy stored in the inductor L_1_ is delivered to the load. The capacitor C_1_ is recharged from the source. Applying KVL to the circuit shown in Fig. 3b, we get
    3 $$v_{L1} = - v_{o}$$
    4 $$v_{C1} = v_{in}$$
    By applying the volt-second balance condition to the voltage across the inductor (L_1_) from Eqs. (1), (2), and (3), the voltage gain (G) of the proposed converter is given by
    5 $$G = \frac{{v_{o} }}{{v_{in} }} = \frac{D}{1 - D}$$
(II) During the negative half cycle of the input voltage
  The operation principle of the proposed converter is the same as that in the positive half cycle. The inductor L_1_ stored energy from the source and the capacitor C_1_ through the path formed by the switch S_2_ and the body diode of S_1_, as shown in Fig. 3c. The energy was then discharged into the load via the path formed by the switch S_3_ and the body diode of S_4_, as shown in Fig. 3d.

(I) During the positive half cycle of the input voltage:
  - Mode 1 [0-DT_s_]:
    Switch S_1_ is turned-on during the DT_s_ interval, as shown in Fig. 3a, and the body diode of S_2_ is forward biased. This formed a path for the capacitor C_1_ to discharge its stored energy through the inductor L_1_. The energy is stored in the inductor L_1_ from the input source and the capacitor C_1_. Applying KVL to the circuit shown in Fig. 3a in steady state operation, we get
    1 $$v_{L1} = v_{C1}$$
    2 $$v_{C1} = v_{in}$$
    where $v_{in}$ represents the input voltage and the voltage drop across the input filter inductor L_in_ is neglected.

## Parameter’s design of the proposed converter

The passive components are mainly designed by considering their maximum tolerable current and voltage ripples. The inductor current ripple and capacitor voltage ripple can be obtained from the following equations:

6 $$v_{l} = L \frac{{\Delta i_{l} }}{\Delta t}$$

7 $$i_{c} = C{ }\frac{{\Delta v_{c} }}{\Delta t}{ }$$

The inductor maximum tolerable current ripple is taken as a factor α% from its maximum rms current $I_{l - rms}^{max}$.

8 $$\Delta I_{L - max} = \alpha { }I_{l - rms}^{max} { }$$

Substituting by Eqs. (1), and (8) in Eq. (6), and for maximum inductor current $I_{l - rms}^{max} = \frac{{I_{o - rms} }}{1 - D}$ the inductor equation will be as follows:

9 $$L_{1} = V_{in - rms} \frac{{D T_{s } \left( {1 - D} \right)}}{{\alpha I_{o - rms} }}$$

10 $$L_{1} = D^{2} \frac{{{ }V^{2}_{in - rms} }}{{\alpha f_{sw} { } P_{{o{ }}} }}$$

The maximum allowable voltage ripple for the capacitor C_1_ is taken as a factor that is defined β% of the peak voltages across it $(\Delta v_{c} = \beta v_{c} )$. Substituting by Eq. (4) in (7) for an interval (1-D)T_s_.

11 $$C_{1} = \frac{{{ }I_{in} \left( {1 - D} \right)}}{{f_{sw } \beta V_{in - rms} }}$$

Considering an ideal circuit then, the capacitor C_1_ can be obtained as

12 $$C_{1} = \frac{{\left( {1 - {\text{D}}} \right){ }P_{{o{ }}} }}{{\beta { }f_{sw} { } V^{2}_{in - rms} }}$$

where *I*_*o-rms*_ is the rms value of the load current, $P_{o }$ is the output power, and $f_{sw}$ is the switching frequency.

For selecting the required ratings of the semiconductor switches of the proposed converter, the peak voltages and currents of the semiconductor switches are calculated from Eqs. (13) and (14).

13 $$V_{{s1 - s4{ }\left( {pk} \right)}} = \sqrt 2 { }\left( {V_{in - rms} + V_{o - rms} } \right)$$

14 $$I_{{s1 - s4{ }\left( {pk} \right)}} = \sqrt {2{ }} \left( {I_{in - rms} + I_{o - rms} } \right) = \sqrt 2 { }\frac{{I_{o - rms} }}{1 - D}$$

## Calculation of power losses and efficiency

### Power losses calculation

Conduction losses, switching losses, and blocking losses are the three types of power losses in any semiconductor switch (IGBT or diode). The blocking losses are low compared to the other two parts and can be neglected^33^.

#### Conduction losses

The instantaneous conduction losses in the IGBT (P_cond.IGBT_) are obtained by multiplying the ON state voltage of the switch and the current following through it.

15 $$\begin{aligned} P_{cond.IGBT} \left( {\text{t}} \right) = & \,\left[ {V_{CE0} + R_{{\text{C}}} . i\left( t \right)} \right] i\left( t \right) \\ = & \,{ }V_{CE0} { }.i\left( t \right) + { }R_{{\text{C}}} { }.i\left( t \right)^{2} \\ \end{aligned}$$

where V_CE0_ is the zero-current collector-emitter voltage during on-state, R_C_ is the collector-emitter resistance during on-state, and i(t) is the current following through the IGBT that equals the inductor current (i_L1_).

There is only one IGBT operating in each mode of operation, so the total conduction losses for the four IGBTs are equivalent to the conduction losses of a single IGBT if it is continuously operating during the full cycle. The average value of the conduction losses can be given by the integration over a half of the periodic time, as the positive and negative half cycles are similar. The IGBT current is the same as the inductor current (i_L1_), thus the average value of conduction losses can be given as:

16 $$\begin{aligned} P_{cond.IGBT avg.} = & \frac{1}{\pi }\mathop \smallint \limits_{0}^{\pi } \left[ {V_{CE0} { }.i\left( t \right) + { }R_{{\text{C}}} { }.i\left( t \right)^{2} } \right]d\left( {wt} \right) \\ = & \,V_{CE0} { }.I_{avg} + { }R_{{\text{C}}} { } \cdot I^{2}_{rms} \\ \end{aligned}$$

where I_*avg*_, I_*rms*_ are the average and RMS values of the switch current. Similarly, the average conduction losses of the diode are given as:

17 $$P_{cond.D. avg.} = V_{D0} { } \cdot I_{avg} + { }R_{{\text{D}}} { } \cdot I^{2}_{rms}$$

where V_D0_ is the diode zero-current voltage, R_D_ is the diode on-state resistance.

The current path is formed by only one IGBT and the body diode of another one during each mode of operation. Therefore, the total conduction losses can be expressed as the sum of the conduction losses for one IGBT and one diode given by Eqs. (16) and (17), respectively.

18 $$P_{cond.total} = { }P_{cond.IGBT avg.} + P_{cond.D. avg.}$$

#### Switching losses

The switching losses of the switch (*P*_*sw*_) can be expressed as:

19 $$P_{sw} = \left( {W_{{{\text{on}}}} + {\text{W}}_{off} } \right) \cdot f_{{{\text{sw}}}}$$

where W_on_ and W_off_ are the energy dissipated during turn-on and turn-off times, respectively^34^. There are two switches that operate respectively during each half cycle, the average switching losses for the proposed converter equal to the sum of the switching losses for the two switches considering that they are turned on and off along the overall cycle.

20 $$P_{sw. avg} = 2 \cdot \left( {P_{sw} } \right)$$

### Passive components power losses calculation

The power losses in the input inductor (L_in_), the main inductor (L_1_), and the main capacitor (C_1_) can be calculated as:

21 $$P_{{L{ }.in}} = { }R_{{{\text{L}}.{\text{in}}}} \cdot I^{2}_{in - rms}$$

22 $$P_{L1} = { }R_{{{\text{L}}1}} { } \cdot I^{2}_{L1 - rms}$$

23 $$P_{C1} = { }R_{{{\text{C}}1}} { } \cdot I^{2}_{C1 - rms}$$

where R_L.in_, R_L1_, and R_C1_ are the internal resistance of the input inductor (L_in_), inductor (L_1_), and the capacitor (C_1_), respectively.

The total power losses in the passive elements are expressed as:

24 $$P_{passive.losses} = { }P_{{L{ }.in}} + P_{L1} + P_{C1}$$

### Converter efficiency

The converter output power can be expressed as:

25 $$P_{o} = { }R_{{\text{o}}} \cdot I^{2}_{o - rms}$$

The converter power losses are the summation of the losses in the switches and passive component losses given by Eqs. (18), (20), and (24).

26 $$P_{Converter \,losses} = { }P_{cond.total} + P_{sw. avg} + P_{passive.losses}$$

The converter input power can be expressed as:

27 $$P_{in} = { }P_{o} + P_{Converter \,losses}$$

The percentage efficiency of the converter can be calculated from Eqs. (25) and (27) as:

28 $$Efficiency {{\%}} = \frac{{P_{o} }}{{P_{in} }} \times 100$$

### Calculation of input power factor

The input power factor (PF) can be obtained as:

29 $$ PF = \frac{{P_{in} }}{{V_{in - rms}} {I_{in - rms}} }$$

## Simulation results

The model of the proposed converter is developed and simulated in the MATLAB/Simulink environment. The circuit performance is evaluated at switching frequency *f*_*sw*_ = 2 kHz with converter parameters summarized in Table 1.

**Table 1:** Proposed converter parameters at *f*_*sw*_ = 2 kHz.

Parameters	Value
Capacitor (*C*_1_)	7 µF
Inductor (*L*_1_)	4.64 mH
Input inductor (*L*_*in*_)	4 mH
Output capacitor (*C*_*out*_)	10 µF
Input voltage (*V*_*in*_)	50 V_rms_/50 Hz
Resistive load (*R*_*o*_)	50 Ω
Inductive load (*R*_*o*_ and *L*_*o*_)	50 Ω & 100 mH

According to Eq. (5), the proposed converter offers a boost operation when D is greater than 0.5, and a buck operation when D is lower than 0.5. The suggested converter was designed and tested at a 2 kHz switching frequency with the parameters shown in Table 1 as it was designed with the available components in the laboratory.

### Simulation results for Resistive load

The system is powered by a 50 V AC supply and is connected to a resistive load (R_o_ = 50 Ω).

**Figure 4:**
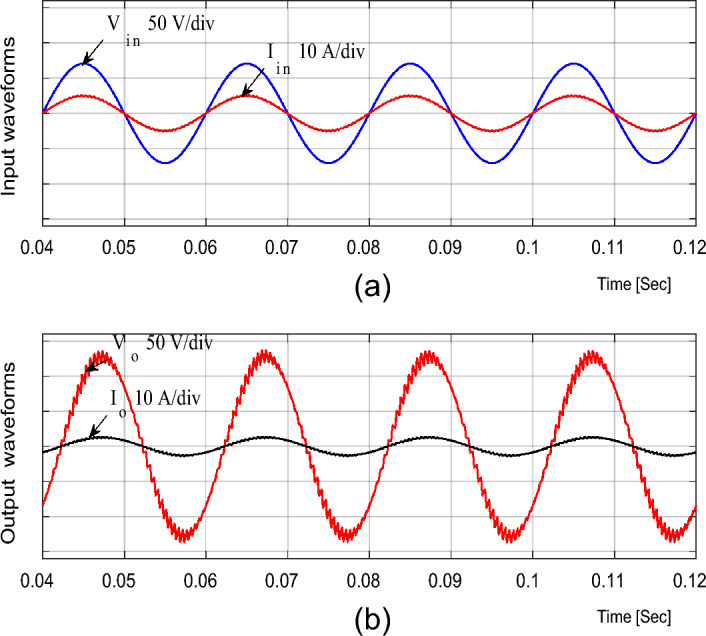
Simulation results of the proposed converter at D = 0.65 and f_sw_ = 2 kHz feeding a resistive load. (**a**) Input voltage and current. (**b**) Output voltage and current.

**Figure 5:**
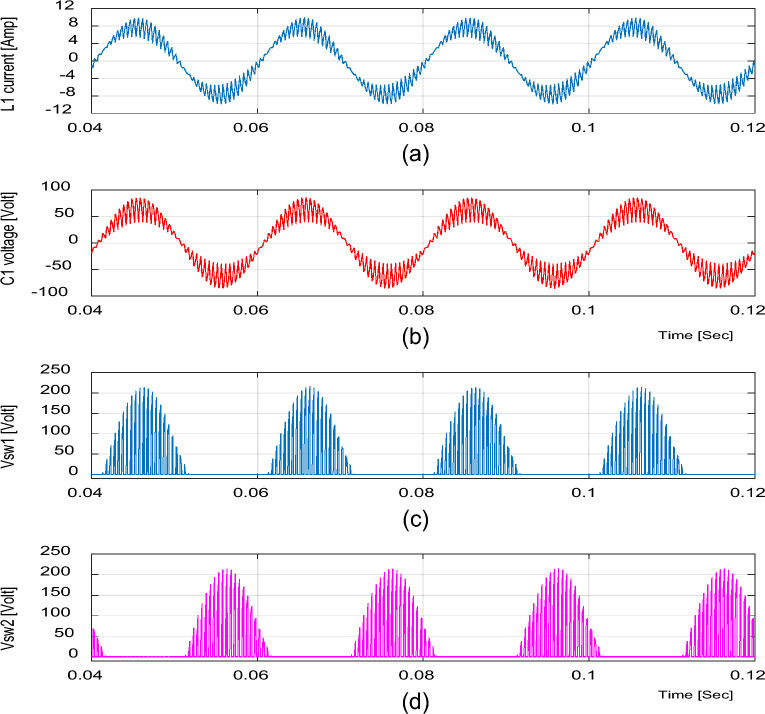
Simulation results of the proposed converter at D = 0.65 and f_sw_ = 2 kHz feeding a resistive load. (**a**) Inductor current (i_L1_). (**b**) Capacitor voltage (v_C1_). (**c**, **d**) Voltage stresses across S_1_ and S_2_.

For boosting mode, the duty ratio is set to 0.65; the input voltage, output voltage, input current, and output current are shown in Fig. 4. It is illustrated that the output voltage equals 92.86 V at an input voltage of 50 V with a voltage gain of 1.857. Moreover, the voltage and current waveforms are continuous. The inductor current (i_L1_), capacitor voltage (*v*_C1_), and voltage stresses across S_1_ and S_2_ at D = 0.65 are shown in Fig. 5. The maximum voltage across the switches S_1_ and S_2_ nearly equals 200 V.

**Figure 6:**
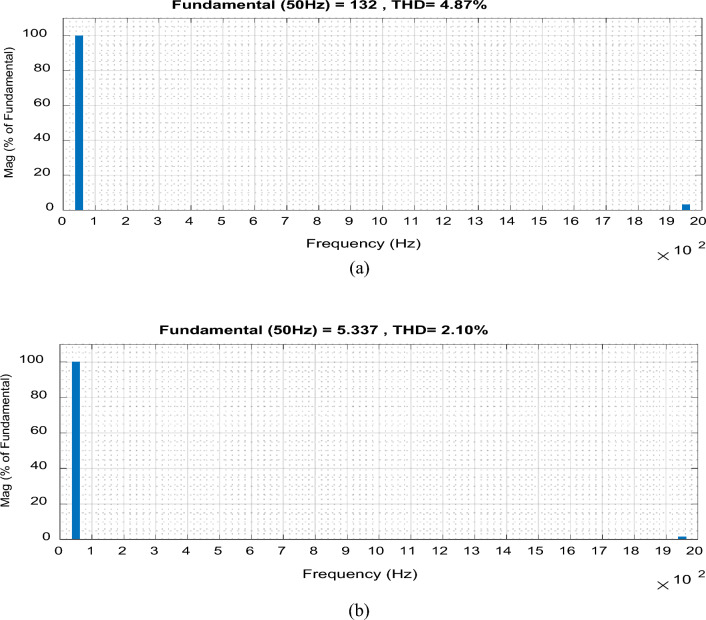
THD for the proposed converter at f_sw_ = 2 kHz. (**a**) Output voltage. (**b**) Input current.

The Total Harmonic Distortion (THD) of the output voltage and the input current are demonstrated, respectively, as shown in Fig. 6. The value of THD for the output voltage is 4.87% and 2.1% for the input current, which are acceptable limits.

**Figure 7:**
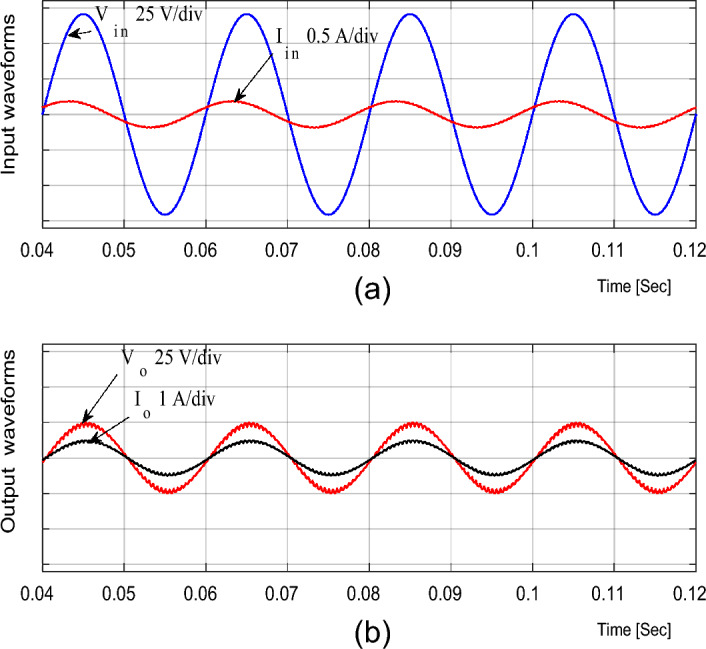
Simulation results of the proposed converter at D = 0.25 and f_sw_ = 2 kHz feeding a resistive load. (**a**) Input voltage and current. (**b**) Output voltage and current.

**Figure 8:**
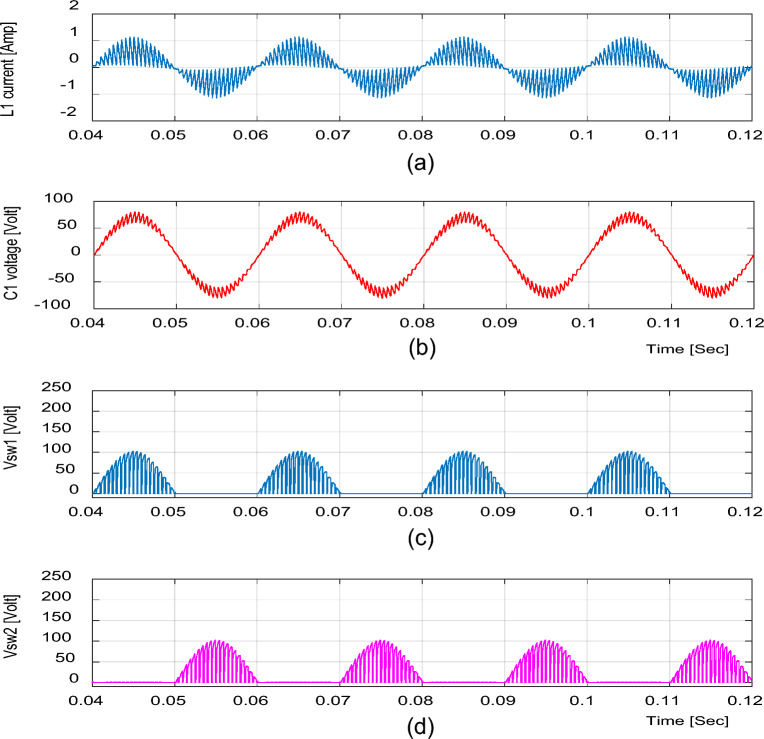
Simulation results of the proposed converter at D = 0.25 and f_sw_ = 2 kHz feeding a resistive load. (**a**) Inductor current (i_L1_). (**b**) Capacitor voltage (v_C1_). (**c**, **d**) Voltage stresses across S_1_ and S_2_.

In bucking mode, the waveforms of the input and output voltages and currents at the duty ratio of 0.25 for an input voltage of 50 V are displayed in Fig. 7. The output voltage is equal to 16.67; thus, the voltage gain equals to 0.33. Figure 8 depicts the inductor current (i_L1_), capacitor voltage (*v*_C1_), and the voltages across S_1_ and S_2_ at D = 0.25, where the voltage stresses on S_1_ and S_2_ nearly equal 95 V.

It is indicated from Figs. 5 and 8 that the switches voltage’s stress equals the addition of the input and output voltages, as indicated from Eq. (13).

The previous results for the proposed converter and Figs. 4, 5, 6, 7 and 8 indicate the high quality of the input and output voltage and current waveforms with high converter efficiency. They also indicate that the input and output currents are continuous and the THD is within acceptable limits.

### Simulation results for Inductive load

**Figure 9:**
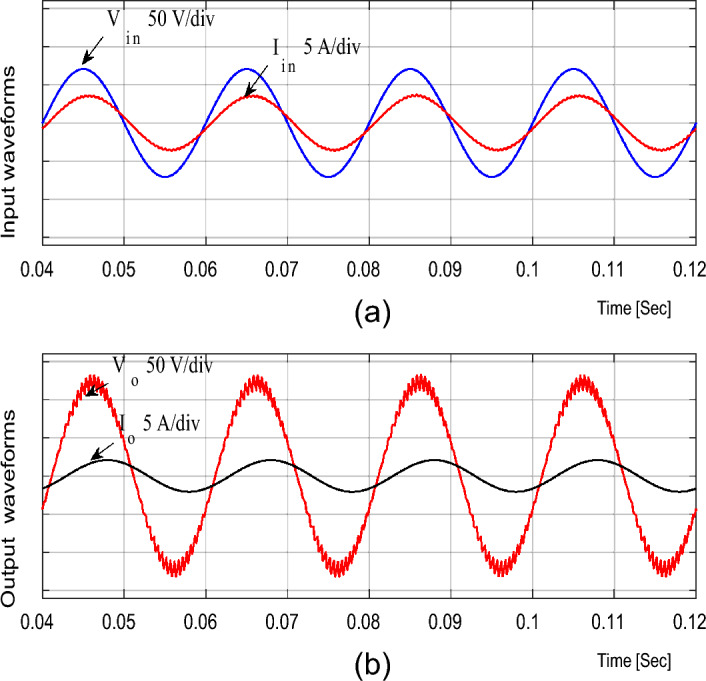
Simulation results of the proposed converter at D = 0.65 and f_sw_ = 2 kHz feeding an inductive load. (**a**) Input voltage and current. (**b**) Output voltage and current.

**Figure 10:**
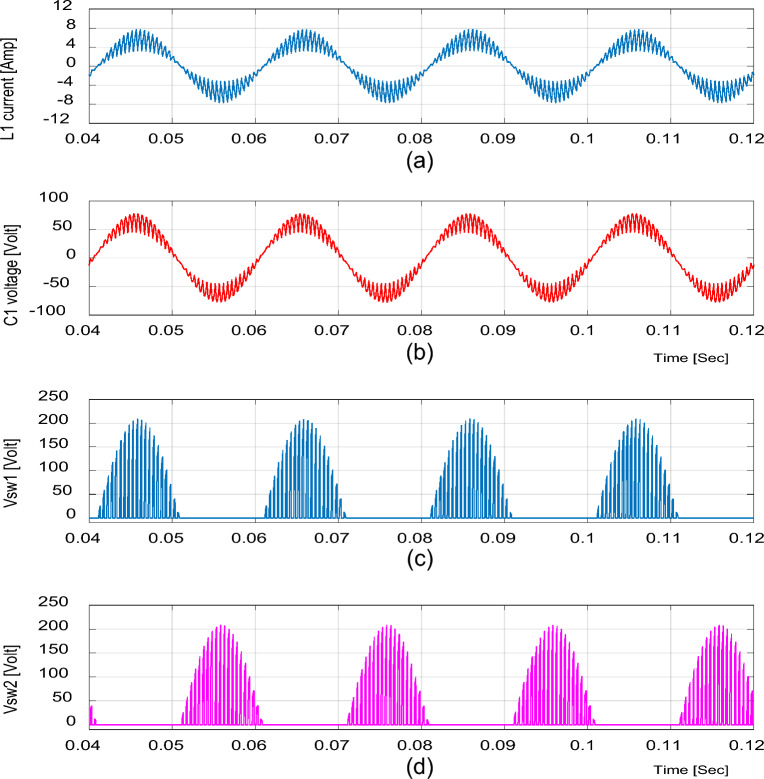
Simulation results of the proposed converter at D = 0.65 and f_sw_ = 2 kHz feeding an inductive load. (**a**) Inductor (L_1_) current. (**b**) Capacitor (C_1_) voltage. (**c**, **d**) Voltage stresses across S_1_ and S_2_.

**Figure 11:**
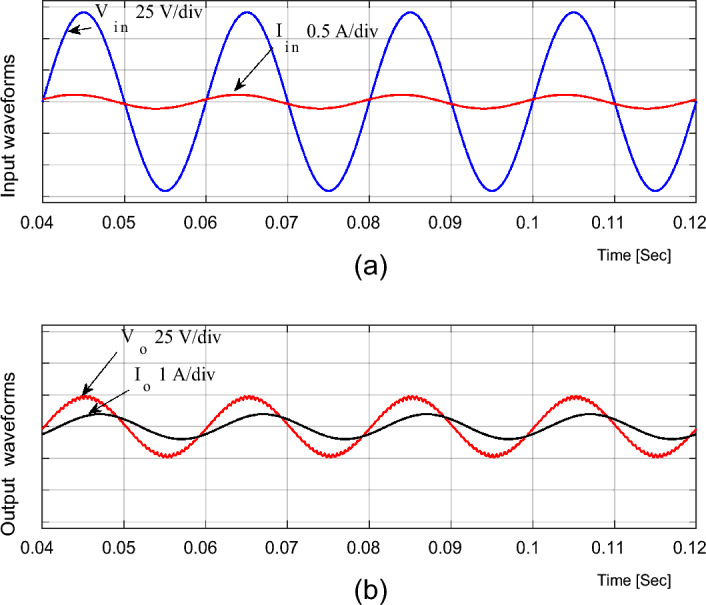
Simulation results of the proposed converter at D = 0.25 and f_sw_ = 2 kHz feeding an inductive load. (**a**) Input voltage and current. (**b**) Output voltage and current.

The simulation results for the proposed converter when supplied by a 50 V AC supply and connected to an inductive load (R_o_ = 50 Ω and *L*_*o*_ = 100 mH) are shown in Figs. 9, 10 and 11.

Figure 9 presents the input and output voltage and current waveforms for boosting mode at D = 0.65. It is obvious that the input and output currents are semi-continous and nearly pure sinsouidal waveforms with low THD. The THD for input and output currents equals 2.16% and 0.3%, respictively.

The output voltage for the inductive load case is the same as for the resistive load, and it has the same voltage gain about 1.857.

Also, Fig. 10 indicates that the inductor current (i_L1_), capacitor voltage (*v*_C1_), and the voltage stresses across S_1_ and S_2_ are nearly the same as with the resistive load.

The performance of the proposed converter with the inductive load is investigated in bucking mode and the simulation results are given in Fig. 11. The supply current is nearly in-phase with the supply voltage; therefore, the supply power factor is nearly unity.

**Figure 12:**
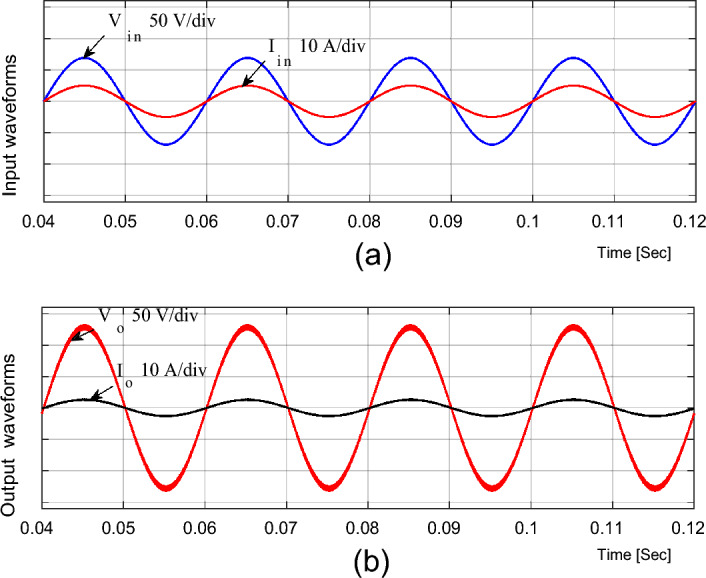
Simulation results of the proposed converter at D = 0.65 and f_sw_ = 60 kHz feeding a resistive load. (**a**) Input voltage and current. (**b**) Output voltage and current.

**Figure 13:**
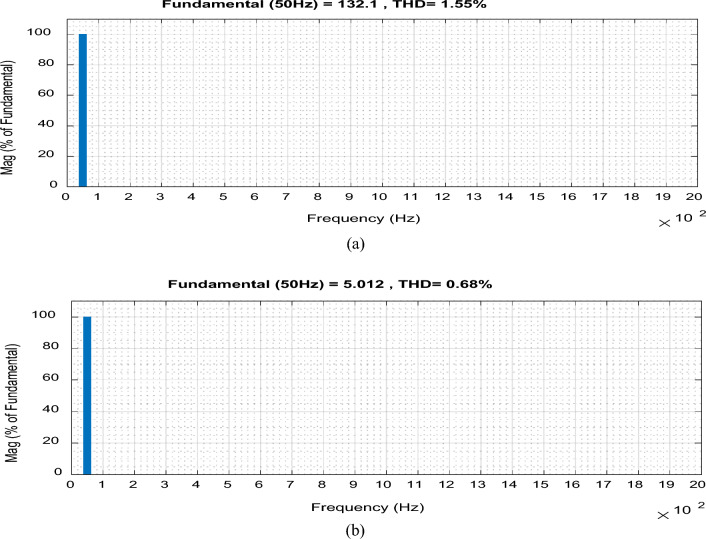
THD for the proposed converter at f_sw_ = 60 kHz. (**a**) Output voltage. (**b**) Input current.

The proposed converter is redesigned at a higher switching frequency (*f*_*sw*_ = 60 kHz) with components listed in Table 2 to achieve higher performance with a smaller size and lower filtering requirements. It will offer high performance with high efficiency, reaching more than 97%. Also, its size will be very small, and the required input filter inductor and output filter capacitor will be very small, as given in Table 2. Figure 12 shows the input and output voltage and current waveforms when the duty ratio is set to D = 0.65 and *f*_*sw*_ = *60 kHz* . The converter offers an output voltage with voltage gain of G = 1.86. The converter efficiency equals 97% and the input power factor (P.F.) equals 0.9997 at D = 0.65. Also, the THD of the output voltage and input current is very low as they equal 1.55% and 0.68%, respectively, as shown in Fig. 13.

**Table 2:** Proposed converter parameters at *f*_*sw*_ = 60 kHz.

Parameters	Value
Capacitor (*C*_1_)	1.5 µF
Inductor (*L*_1_)	0.45 mH
Input inductor (*L*_*in*_)	0.5 mH
Output capacitor (*C*_*out*_)	2 µF
Input voltage (*V*_*in*_)	50 V_rms_/50 Hz
Resistive load (*R*_*o*_)	50 Ω
Inductive load (*R*_*o*_ and *L*_*o*_)	50 Ω and 100 mH

Figures 12 and 13 indicate the high quality of the input and output voltages and currents waveforms. They also indicate the continuity of the input and output current waveforms and a low THD with a minimum passive component size.

## Comparison between various AC–AC converters

A comparison of the proposed buck-boost AC–AC converter with some of the recent direct AC–AC converters is given in Table 3. The comparison is given in terms of the number of switches, power diodes, passive components, and switches operating at high switching frequency in each mode of operation. Based on the comparison in Table 3, it is clear that the proposed converter is designed with a minimum number of switches, and a minimum number of passive components than the competitor. Reducing the power electronics components means reducing the size, the total power losses, and the total cost of the converter.

**Table 3:** Comparison between various AC–AC converters.

Description	Proposed converter	AC–AC converter of^25^	AC–AC converter of^26^	AC–AC converter of^27^	AC–AC converter of^28^	AC–AC converter of^29^	AC–AC converter of^30^
No. of switches	4 (S_1_–S_4_)	4 (S_1_–S_4_)	4 (S_1_–S_4_)	4 (S_1_–S_4_)	6 (S_1_–S_6_)	6 (S_1_–S_6_)	6 (S_1_–S_6_)
No. of diodes	–	4 (D_1_–D_4_)	4 (D_1_–D_4_)	4 (D_1_–D_4_) 6 (D_z1_–D_z6_)	–	–	6 (D_1_–D_6_)
No. of inductors	1 (L_1_)	5 (L_1_–L_4_, L)	2 (L_1_–L_2_)	4 (L_1_– L_4_)	2 (L_1_–L_2_)	2 (L_in_, L_o_)	1 (L_1_)
No. of energy storing or bypass capacitors	1 (C_1_) 1 (C_out_)	2 (C_1_–C_2_) 2 (C_in_, C_out_)	2 (C_1_–C_2_) 2 (C_in_, C_out_)	2 (C_1_–C_2_) 2 (C_in_, C_out_)	2 (C_1_, C_2_) 1 (C_out_)	1 (C_1_)	2 (C_in_, C_out_)
Commutation problem	No	No	No	No	No	No	No
Need soft commutation strategy	No	Yes	No	No	No	Yes	Yes
Total No. of switches operating with high switching frequency in each switching cycle	1	8	8	7	2	3	2
Max. No. of conducting semiconductors among modes of operation	2	4	4	4	3	4	4
Continuity of input and output currents	Continuous	Quasi-continuous	Quasi-continuous	Quasi-continuous	Quasi-continuous	Quasi-continuous	Quasi-continuous
Required input/output filters	Small L_in_ Small C_out_	Moderate L_in_ and C_in_ Moderate C_out_	Moderate L_in_ and C_in_ Moderate C_out_	Moderate L_in_ and C_in_ Moderate C_out_	Small L_in_ Small C_out_	Small L_in_ and C_in_ Moderate C_out_	Moderate C_in_ Moderate C_out_
Voltage gain $\frac{{v_{o} }}{{v_{in} }}$	$\frac{D}{{1 - {\text{D}}}}$	$\frac{D}{{1 - {\text{D}}}}$	$\frac{D}{{1 - {\text{D}}}}$	$\frac{2D}{{1 - {\text{D}}}}$	D/$\frac{D}{{1 - {\text{D}}}}$	$\frac{D}{{1 - {\text{D}}}}$	D

## Experimental results

**Figure 14:**
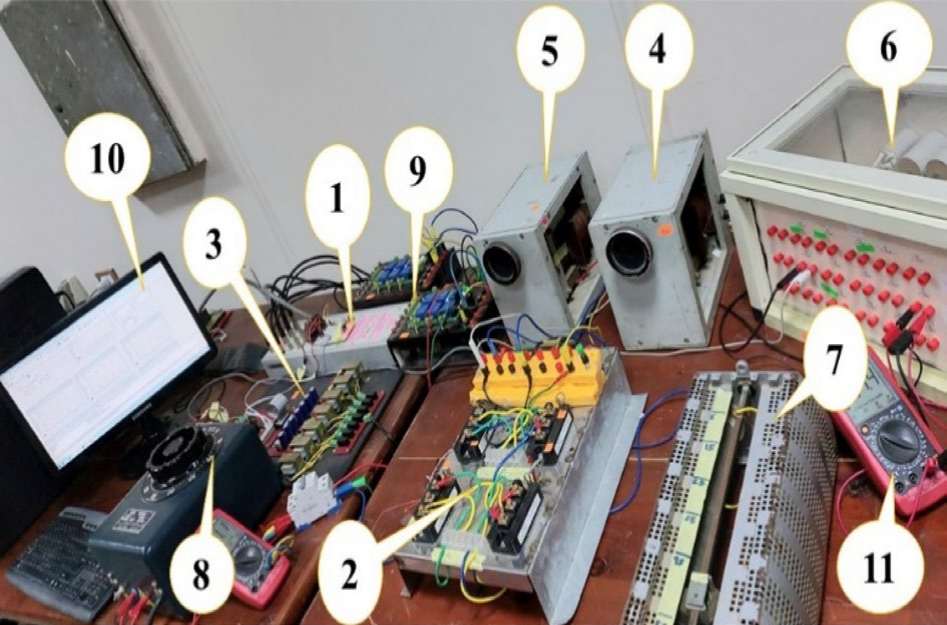
Experimental setup for the proposed converter.

The proposed converter is investigated in the laboratory to verify the aimed circuit. An experimental setup is implemented with components listed in Table 4, as shown in Fig. 14. The control system used for generating the gating signals of the controlled switches is a DSP-based laboratory model. Then, a drive circuit is used to amplify the voltage of the IGBTs pulses taken from the dSPACE (DS-1104) platform and to isolate the control system from the power system. The drive circuit can operate with a maximum frequency limit of 2 kHz due to the DSP limitations, so the experimental results are obtained at *f*_*sw*_ = 2 kHz based on the sampling rate of the control board. The electrical specifications of the prototype at *f*_*sw*_ = 2 kHz are given in Table 1. The active switches (S_1_-S_4_) are represented by two modules of MITSUBSHI CM100DY-24H IGBTs.

**Table 4:** Experimental components.

No	Component	No	Component
1	dSPACE (DS1104) platform	7	Load resistor (R_o_)
2	2 IGBTs modules	8	Autotransformer
3	Drive circuit	9	Transducer kit
4	Inductor (L_1_)	10	Computer
5	Input filter inductor (L_in_)	11	Measurement device
6	Capacitors box (C_1_ and C_out_)

A signal transducer’s kits are used to measure the circuit current via the LA25-NP current sensor and the circuit voltages via the LV25-P voltage sensors. The measured currents and voltages are sent to dSPACE (DS-1104) platform via the DSP Analogue to Digital interface.

### Experimental results for resistive load

**Figure 15:**
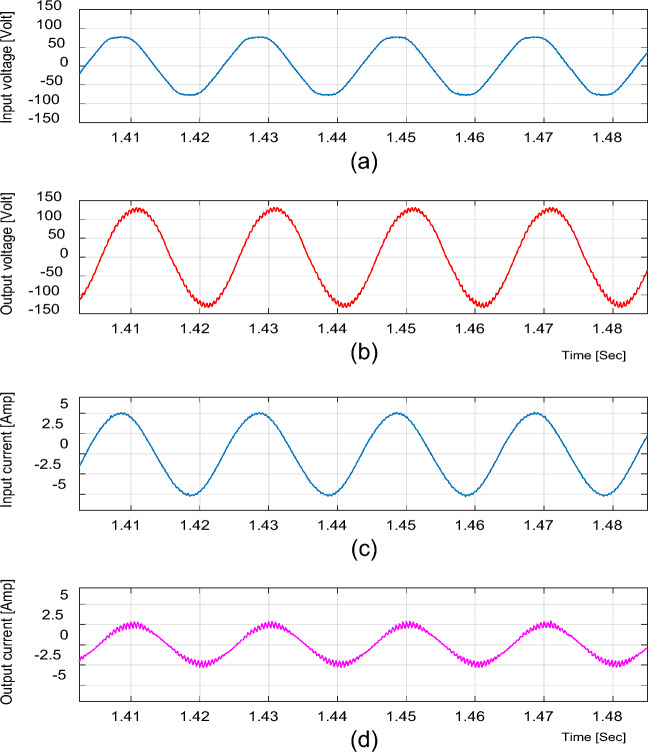
Experimental results of the proposed converter at D = 0.65 and f_sw_ = 2 kHz feeding a resistive load. (**a**) Input voltage. (**b**) Output voltage. (**c**) Input current. (**d**) Output current.

**Figure 16:**
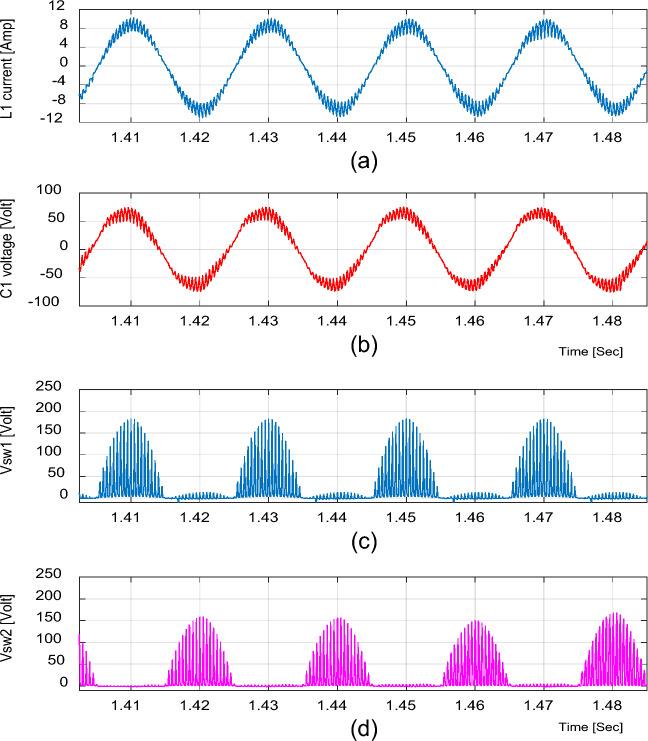
Experimental results of the proposed converter at D = 0.65 and f_sw_ = 2 kHz feeding a resistive load. (**a**) Inductor current (i_L1_). (**b**) Capacitor voltage (v_C1_). (**c**, **d**) Voltage stresses across S_1_ and S_2_.

**Figure 17:**
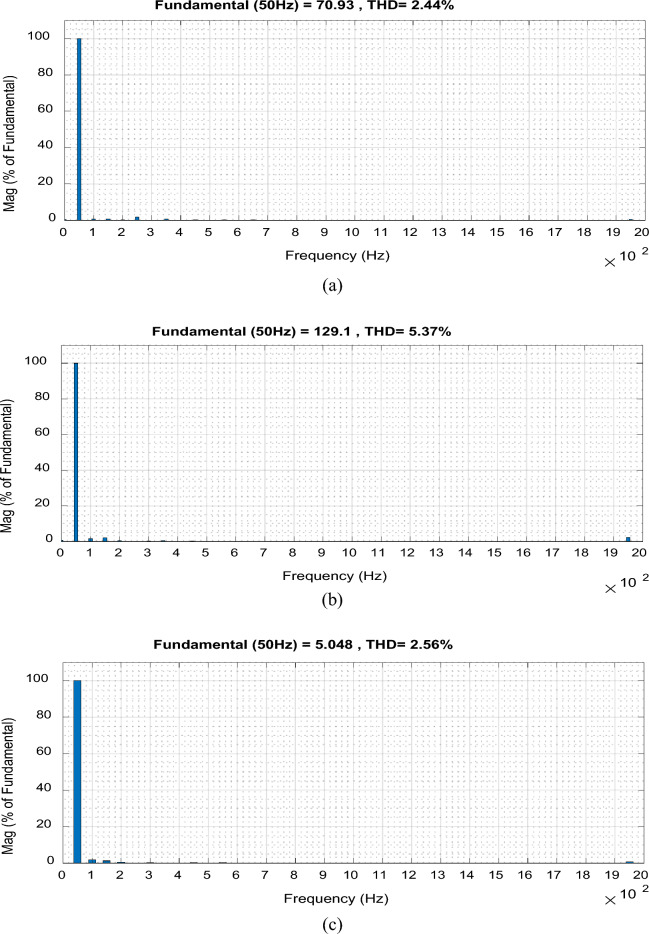
THD for the proposed converter at f_sw_ = 2 kHz. (**a**) Input voltage. (**b**) Output voltage. (**c**) Input current.

**Figure 18:**
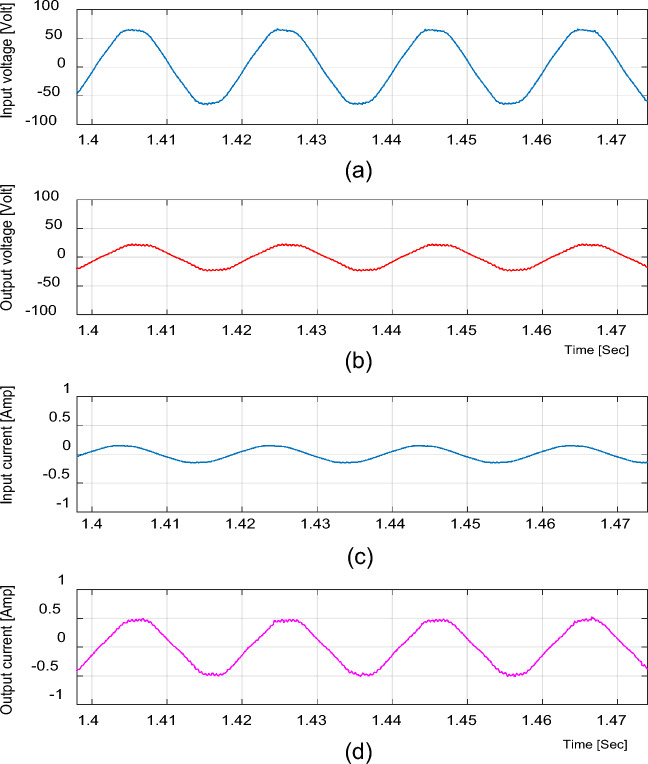
Experimental results of the proposed converter at D = 0.25 and f_sw_ = 2 kHz feeding a resistive load. (**a**) Input voltage. (**b**) Output voltage. (**c**) Input current. (**d**) Output current.

**Figure 19:**
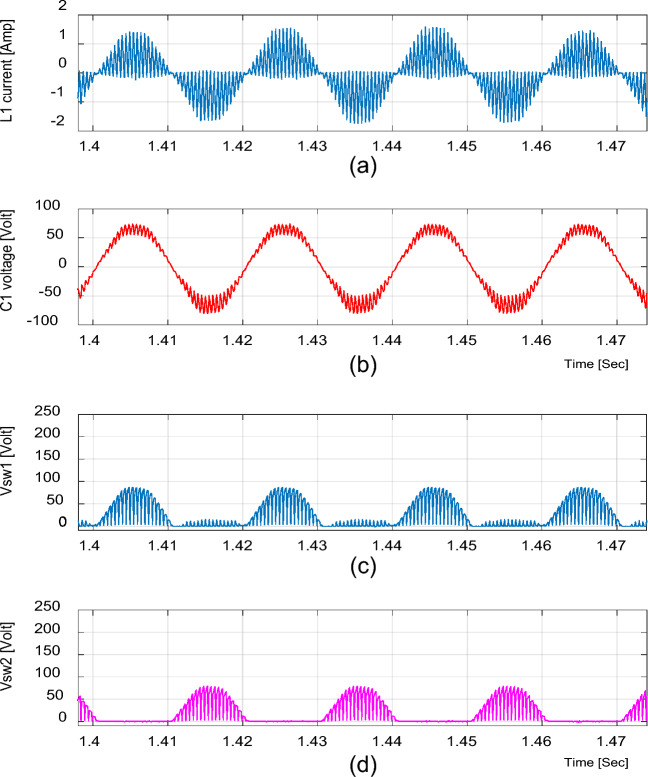
Experimental results of the proposed converter at D = 0.25 and f_sw_ = 2 kHz feeding a resistive load. (**a**) Inductor current (i_L1_). (**b**) Capacitor voltage (v_C1_). (**c**, **d**) Voltage stresses across S_1_ and S_2_.

For the resistive load, the experimental input and output voltage and current waveforms for boost and buck operations are shown in Figs. 15, 16, 17, 18 and 19. From these figures, it can be seen that the measured voltage gain is closer to the theoretical value and simulation results.

For boosting mode, the voltage gain equals 1.789 at D = 0.65. The input and output currents are continuous and nearly pure sinusoidal waves as shown in Fig. 15. It is observed from Fig. 17 that the THD of input voltage, output voltage, and input current are within acceptable limits. At D = 0.65, the converter efficiency equals 92.3% and the input power factor equals 0.995.

For bucking mode, the voltage gain equals 0.309 at D = 0.25 and the input power factor equals 0.9656, as shown in Fig. 18.

Figures 16 and 19 show the inductor current (i_L1_), capacitor voltage (v_C1_), and voltage stresses across S_1_ and S_2_ for boost and buck operation, respectively. As seen from these figures, the experimental results are in good agreement with the simulation results. In addition, the maximum value of the voltage across the switches is in good agreement with the calculated value from Eq. (13).

### Experimental results for inductive load

**Figure 20:**
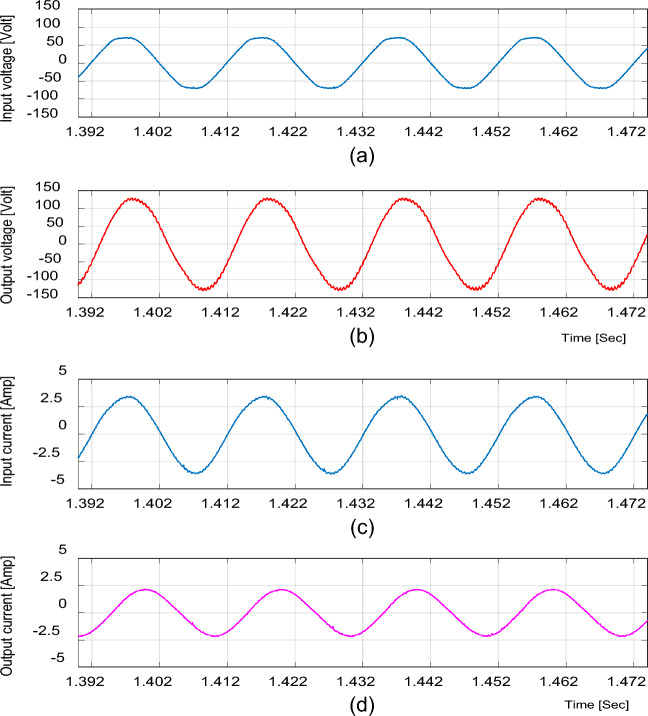
Experimental results of the proposed converter at D = 0.65 and f_sw_ = 2 kHz feeding an inductive load. (**a**) Input voltage. (**b**) Output voltage. (**c**) Input current. (**d**) Output current.

**Figure 21:**
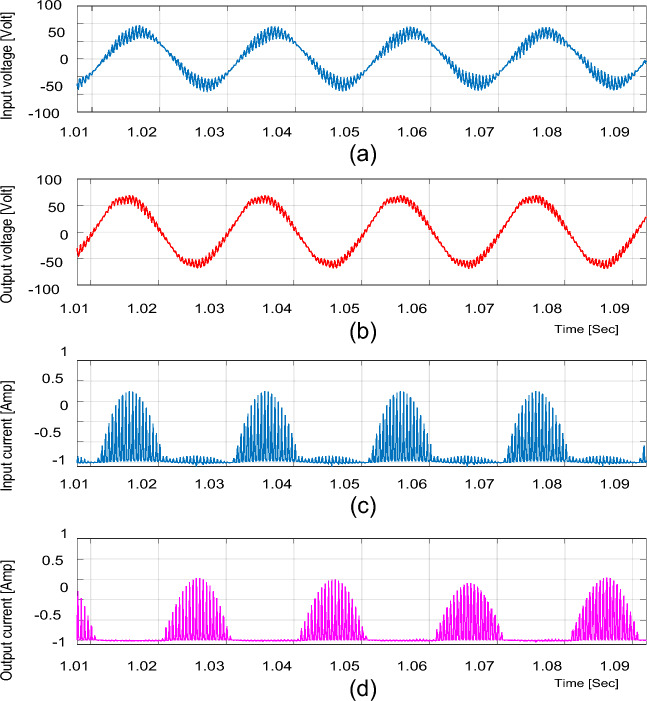
Experimental results of the proposed converter at D = 0.65 and f_sw_ = 2 kHz feeding an inductive load. (**a**) Inductor current (i_L1_). (**b**) Capacitor voltage (v_C1_). (**c**, **d**) Voltage stresses across S_1_ and S_2_.

**Figure 22:**
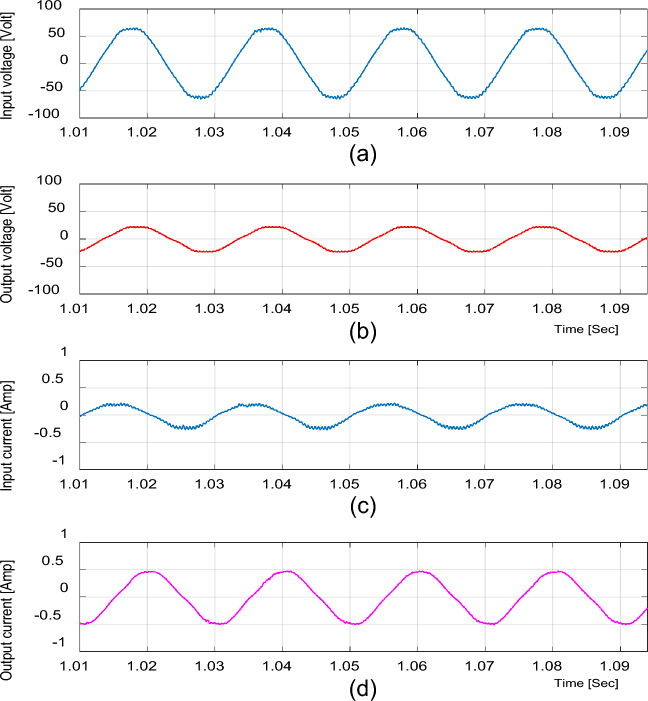
Experimental results of the proposed converter at D = 0.25 and f_sw_ = 2 kHz feeding an inductive load. (a) Input voltage. (**b**) Output voltage. (**c**) Input current.(**d**) Output current.

To validate the ability of the proposed converter to feed the inductive loads, the experimental results are given by the following figures. The input and output voltage and current waveforms at D = 0.65 are shown in Fig. 20. The inductor current (i_L1_), capacitor voltage (v_C1_), and voltage stresses across S_1_ and S_2_ at D = 0.65 are shown in Fig. 21. The input and output voltage and current waveforms at D = 0.25 are shown in Fig. 22.

These figures indicate that the proposed converter operates with the inductive load with high quality nearly sinusoidal waveforms as well as it operates with the resistive load.

**Figure 23:**
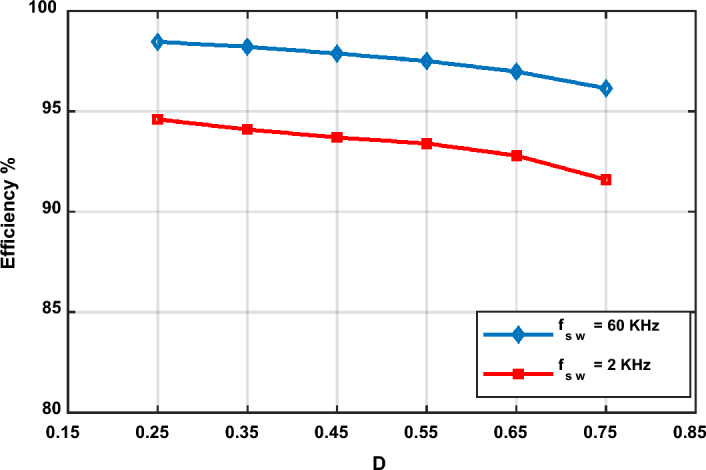
Efficiency of the proposed converter at *f*_*sw*_ = 60 kHz and *f*_*sw*_ = 2 kHz.

The efficiency of the proposed converter when operated with a 60 kHz switching frequency varies with the variation of the duty ratio for almost the same input voltage (50 V-rms) and the same load (R_o_ = 50 Ω), as shown in Fig. 23. The proposed converter has a peak efficiency of 98.46% when the duty ratio is 0.25 and a minimum efficiency of 96.15% when the duty ratio is 0.75.

When the input voltage is 50 V, *f*_*sw*_ is 2 kHz, and R_o_ is 50 Ω, the efficiency of the proposed converter varies with the variation of the duty ratio, as shown in Fig. 23. Experimentally, the proposed converter offers a peak efficiency of 94.6% when the duty ratio is 0.25. The minimum efficiency of the proposed converter is 91.6% when the duty ratio is 0.75. Moreover, for the whole range of duty ratio variations, the efficiency of the proposed converter is more than 91.6% for almost the same input voltage and the same load, as shown in Fig. 23.

As noticed from Fig. 23, the efficiency of the proposed converter when operated with 60 kHz is higher than when operated with 2 kHz due to the large components used in the experimental setup in the laboratory that increase the power losses.

The proposed converter offers a higher efficiency than the converters described in^25^ and^28^ when operated at the same experimental conditions as the maximum efficiency achieved with the two converters are 95.2% and 96.8%, respectively. The high efficiency of the proposed converter is the result of the lower number of semiconductor switches and passive components utilized in the proposed converter.

## Conclusion

This paper introduced a direct buck-boost AC–AC converter with a low count of semiconductor switches with a lower rating and a minimum number of passive components. Thus, the converter size and the power losses are decreased, and the converter efficiency increases. The switching algorithm is discussed and is very simple. The parameters’ design procedures and circuit analysis were detailed. A comparative study with previous converters was carried out, indicating that the proposed converter is superior to other converters. The proposed topology has been verified via a simulation assessment and an experimental setup for different conditions. The THDs for the input and output waveforms are within acceptable limits. An excellent agreement is found between simulation and experimental results, achieving the suggested circuit. The circuit structure may be of special interest for voltage sag and swell compensation for improving the performance of power systems.

## Data Availability

The datasets used and/or analyzed during the current study available from the corresponding author on reasonable request.
